# Training with reduced carbohydrate availability affects markers of bone resorption and formation in male academy soccer players from the English Premier League

**DOI:** 10.1007/s00421-024-05574-4

**Published:** 2024-08-18

**Authors:** Reuben Stables, Liam Anderson, Craig Sale, Marcus P. Hannon, Rachel Dunn, Jonathan C. Y. Tang, William D. Fraser, Nessan B. Costello, Graeme L. Close, James P. Morton

**Affiliations:** 1 Research Institute for Sport and Exercise Sciences (RISES), Liverpool John Moores University, Byrom Street, Liverpool, L3 3AF UK; 2 School of Sport, Exercise and Rehabilitation Sciences, University of Birmingham, Birmingham, UK; 3 Department of Sport and Exercise Sciences, Manchester Metropolitan University Institute of Sport, 99 Oxford Road, Manchester, UK; 4 Bioanalytical Facility, Norwich Medical School, University of East Anglia, Norwich, UK; 5 Departments of Clinical Biochemistry, Diabetes and Endocrinology, Norfolk and Norwich University Hospital NHS Foundation Trust, Colney Lane, Norwich, UK; 6 Carnegie Faculty, Institute for Sport, Physical Activity and Leisure, Leeds Beckett University, Leeds, UK

**Keywords:** Bone turnover, Association football, Adolescents

## Abstract

**Purpose:**

To test the hypothesis that training with reduced carbohydrate (CHO) availability increases bone resorption in adolescent soccer players.

**Methods:**

In a randomised crossover design, ten male players (age: 17.4 ± 0.8 years) from an English Premier League academy completed an acute 90-min field-based training session (occurring between 10:30–12:00) in conditions of high (TRAIN HIGH; 1.5 g.kg^−1^, 60 g, 1.5 g.kg^−1^ and 1.5 g.kg^−1^ consumed at 08:00, during training, 12:30 and 13:30, respectively) or low CHO availability (TRAIN LOW; 0 g.kg^−1^). Participants also completed a non-exercise trial (REST) under identical dietary conditions to TRAIN LOW. Venous blood samples were obtained at 08:30, 10:30, 12:30 and 14:30 for assessment of bone resorption (βCTX), bone formation (PINP) and calcium metabolism (PTH and ACa).

**Results:**

External training load did not differ (all* P* > 0.05) between TRAIN HIGH and TRAIN LOW, as evident for total distance (5.6 ± 0.8; 5.5 ± 0.1 km), average speed (81 ± 9; 85 ± 12 m.min^−1^) and high-speed running (350 ± 239; 270 ± 89 m). Area under the curve for both βCTX and PINP was significantly greater (*P* < 0.01 and *P* = 0.03) in TRAIN LOW versus TRAIN HIGH, whilst no differences in PTH or ACa (*P* = 0.11 and *P* = 0.89) were observed between all three trials.

**Conclusion:**

CHO restriction before, during and after an acute soccer training session increased bone (re)modelling markers in academy players. Despite acute anabolic effects of bone formation, the long-term consequence of bone resorption may impair skeletal development and increase injury risk during growth and maturation.

## Introduction

The purpose of soccer academies is to develop the technical, tactical, physical, and psychosocial capabilities of young players (Wrigley et al. 2012). Within the English academy system, players are exposed to a formalised and structured coaching programme whereby they transition through distinct development phases, that is, the Foundation Phase (FP: under 9–11 years old), Youth Development Phase (YDP: under 12–16 years old) and Professional Development Phase (PDP: under 17–23 years old). In relation to physical development, the typical weekly training volume (e.g. total weekly duration of activity and distance covered) that players are exposed to increases as they progress through each development phase (Hannon et al. 2021a). In addition, academy players experience similar absolute training volumes (Brownlee et al. 2018; Hannon et al. 2021a; Stables et al. 2023) as their adult counterparts from the English Premier League (EPL) (Anderson et al. 2016a), albeit it a time when they are not yet fully mature. In using the doubly labelled water method, we also reported that individual players across the academy pathway (*i.e.,* from U12 to U18) may present with an absolute total daily energy expenditure (*i.e.,* 3000 – 5000 kcal.day^−1^) that is comparable to, or exceeds (Hannon et al. 2021b; Stables et al. 2023), our previous observations from adult players of the EPL (Anderson et al. 2017).

Despite such high training volumes and energetic demands, it is often reported that academy players “under-fuel” (*i.e.,* fail to consume sufficient energy and carbohydrate intake), especially in relation to the acute period before, during and after training sessions (Hannon et al. 2021b; Naughton et al. 2016; Stables et al. 2022). Although the negative outcomes associated with “under-fuelling” are often considered from a performance perspective, a more concerning outcome for adolescent athletes is the potential impact upon risk of injury to skeletal structures (Goulding 2007), especially when considering that adolescence is a critical time for bone development (Zhang et al. 2023). In this regard, failing to increase daily energy intake in consideration of the increased resting metabolic rate that accompanies growth and maturation (Hannon et al. 2020) alongside the enhanced energetic cost that is inherent to academy coaching programmes (Stables et al. 2023), may increase the risk of players with presenting chronically low energy availability (LEA) (Mountjoy et al. 2023). In this way, players may subsequently present with negative symptoms associated with LEA, where such symptoms could include reductions in bone accrual. While such conditions may not directly lead to stress fractures alone, under a state of imbalance between microdamage to skeletal tissue formation and breakdown, bone stress injuries may occur. The continual substantial loading to microcracks in the bone under stress therefore presents an increase in stress fracture risk (Hoenig et al. 2023). This is of critical importance for academy soccer players given the prevalence of growth-related injuries to the knee, lower back, sacrum and pelvis, as reported in academy players from England, Europe and South America (Hall et al. 2020).

A growing of body of literature now demonstrates the complex interplay between exercise, nutrient availability, and bone (re)modelling (Dolan et al. 2020). Indeed, we (Hammond et al. 2019) and others (Sale et al. 2015; de Sousa et al. 2014) observed that the mechanical and/or metabolic stress associated with running exercise is sufficient to increase bone resorption in male adults (as evidenced by acute changes in β-carboxyterminal telopeptide, βCTX). Although the greater rates of bone resorption (especially at bony sites) within the adolescent compared to adult population are considered essential to facilitate skeletal development (Zhang et al. 2023), it is noteworthy that the exercise-induced increases in βCTX in adults is significantly reduced if carbohydrate (CHO) has been consumed before, during and/or after exercise (Townsend et al. 2017; Sale et al. 2015; Hammond et al. 2019; de Sousa et al. 2014). Furthermore, when a cohort of male adult racewalkers (Fensham et al. 2022) adhered to a short-term six-day dietary intervention comprising reduced daily CHO intake (*i.e.,* 0.5 g.kg^−1^ CHO, energy availability of 41 kcal·kg FFM^−1^·day^−1^), concentrations of procollagen-1 N-terminal peptide (PINP; a marker of bone formation) were significantly reduced when compared to a control diet matched for energy availability but higher daily CHO intake (*i.e.,* 41 kcal·kg FFM^−1^·day^−1^ and 9.8 g.kg^−1^ CHO) or a diet representative of LEA and moderate daily CHO intake (*i.e.,* 15 kcal·kg FFM^−1^·day^−1^ and 5 g.kg^−1^ CHO per day). When taken together, such data suggest that reductions in both acute (*i.e.,* CHO consumed within several hours of training) and chronic daily CHO intake increases bone resorption the result of which, if persistent over time, might contribute to compromised skeletal development. However, despite the observation that soccer training is considered anabolic to bone (Varley et al. 2023), the acute effects of the habitual soccer training sessions completed by academy players, and the context of such effects within the wider process of acute bone resorption and formation has not yet been evaluated, let alone any potential modulatory role of CHO availability.

With this in mind, the aims of this present study were two-fold. First, we sought to evaluate the effects of an acute soccer training session on markers of bone resorption, bone formation and calcium metabolism in a cohort of male academy soccer players. Second, we also aimed to evaluate the effects of training with reduced CHO availability in modulating markers associated with bone resorption, formation and calcium metabolism. We hypothesised that training with reduced CHO availability (*i.e.,* under-fuelling) would increase markers of bone resorption and reduce markers of bone formation (effects occurring independent of alterations to calcium metabolism).

## Methods

### Participants

Twelve male outfield soccer players from an English Premier League academy volunteered to participate in this study. However, two participants had to withdraw from the study due to pitch-based injuries (not occurring during the training sessions completed as part of this study), leaving ten players who completed all experimental trials (age: 17.4 ± 0.8 yrs; body mass: 74.6 ± 9.1 kg; height: 1.8 ± 0.1 m). On the basis of previous assessments from our laboratory (albeit on adult males) using acute high-intensity intermittent running as an exercise stimulus and a CHO feeding intervention (Hammond et al. 2019), sample size was estimated according to our primary outcome variable of βCTX assuming an effect of CHO availability of 0.3 ng·mL^−1^ and a group standard deviation of 0.2 ng·mL^−1^. These data would provide an effect size of dz = 1.5 where a sample size of 8 would provide an alpha value of 0.05 and statistical power of 0.95 (G* Power, version 3.1). All procedures conformed to the standards of the Declaration of Helsinki, written informed parental / guardian consent and player assent was obtained, and ethical approval was granted by Liverpool John Moores University.

### Study design

**Fig. 1:**
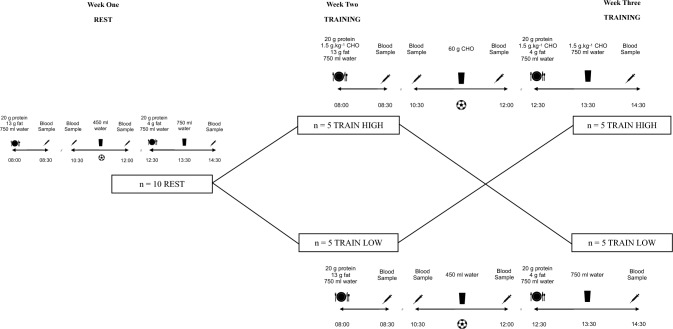
Schematic overview of the experimental design. Participants completed one rest day followed by two experimental trials separated by one week respectively

In a repeated measures (and crossover) design, participants completed three experimental trials that occurred over a 3 week in-season period in April 2023. Trial 1 was a non-exercise trial (REST) that occurred on the Wednesday of week 1 and represented a non-training day for the participants. Trial 2 was a training day that occurred on the Tuesday of week 2 and took place 4 days before the players’ next game (referred to as match day minus 4, MD-4). In trial 2, players were randomised such that half of the sample (n = 5) completed the training session in conditions of high CHO availability (TRAIN HIGH) whilst the remaining participants (n = 5) completed the session in conditions of low CHO availability (TRAIN LOW). Trial 3 occurred on the Tuesday of week 3 (*i.e.,* MD-4) and on this occasion, participants crossed over in trials such that those players who completed trial 2 with high CHO availability now adhered to the low CHO availability trial and vice versa. Players were blinded to CHO availability throughout all trials and completed an overnight fast prior to each trial. To examine the effects of CHO availability upon markers of bone resorption and formation, both REST and TRAIN LOW trials were CHO restricted. An unintended consequence of this is, of course, that these trials were also energy restricted in comparison to TRAIN HIGH. To alleviate this, an option would have been to manipulate the fat and/or protein contents of the dietary intake, but both of these are also known to have independent effects on bone (Walsh and Henriksen 2010). The club coaching staff were instructed to replicate the session duration and content (*i.e.,* training drill content, duration and sequence) during both trial 2 and trial 3 to match the exercise stimulus as closely as possible, similarly all participants completed the same exercise stimulus the day before the TRAIN HIGH and TRAIN LOW trials, although this was not directly controlled within the study. An overview of the experimental design which details dietary intake of each trial is presented in Fig. 1. Further details of the dietary trials and experimental protocols are provided in Table 1.

**Table 1:** Dietary protocol adhered to by participants during the TRAIN HIGH, TRAIN LOW and REST trials

	TRAIN HIGH	TRAIN LOW	REST
Energy (kcal)	1733 ± 163	380	380
Carbohydrate (g)	396 ± 41	0	0
Protein (g)	40	40	40
Fat (g)	17	17	17
Fluid (L)	2.7	2.7	2.7
Calcium (mg)	75	75	75

Mean (± standard deviation) energy (kcal) and carbohydrate (g) is reported alongside the standardised protein (g), fat (g), fluid (L) and calcium intake (mg). In relative terms, CHO intake corresponded to 5.3 ± 0.1 g.kg^−1^ body mass

### Experimental protocols

For all trials, participants reported to the training ground of the host club at 08:00 in a fasted state. After baseline assessment of body mass, (SECA, Hamburg, Germany), participants subsequently consumed breakfast (details for each trial provided below) and an initial venous blood sample was then obtained at 08:30. Due to limitations of the number of samples that could be taken and a lack prior access to participants, no fasted blood sample could be obtained. Further venous blood samples were collected at subsequent 2-h intervals, corresponding to 10:30, 12:30 and 14:30.

#### REST trial

During the REST trial, participants remained at the host training ground and took part in light activities only (e.g. performance analysis education sessions, watching television, playing video games and/or playing pool). Participants consumed a 750 mL placebo beverage at breakfast (150 mL of sugar free orange cordial (Robinsons, UK) diluted in 600 mL of water) and a portion of scrambled egg equivalent to approximately 20 g of protein and < 15 g fat. Participants also consumed 3 × 150 mL boluses of the placebo beverage (125 mL boluses of water mixed with 25 ml of sugar free cordial) at 10:30, 10:50 and 11:10, to replicate the pattern of fluid ingestion that would occur during the training sessions to be completed in both the TRAIN LOW and TRAIN HIGH trials. At 12:30, participants then consumed another 750 mL bolus of the placebo solution, a chicken breast (equivalent to approximately 20 g of protein) and small mixed leaf salad (30 g portion with negligible energy). A final 750 mL bolus of the placebo beverage was consumed at 13:30.

#### TRAIN LOW trial

During the TRAIN LOW trial, participants adhered to the same dietary trial as that administered in the REST trial and participants took part in a 90-min field-based training session occurring between 10:30 and 12:00.

#### TRAIN HIGH trial

During the TRAIN HIGH trial, participants adhered to the same order and timing of dietary intake and fluid ingestion (including the consumption of scrambled eggs and chicken / salad at breakfast and post-training), though a high CHO availability trial now occurred. Carbohydrate was consumed at 08:00 (1.5 g.kg^−1^ of maltodextrin added to 600 mL of water and 150 mL of sugar free cordial during training) followed by 60 g during training (equivalent to 3 × 20 g intakes of maltodextrin consumed at 10:30, 10:50 and 11:10, delivered as 3 × 125 mL boluses of water mixed with 25 mL of sugar free cordial). Carbohydrate was also consumed immediately post-training at 12:30 and again at 13:30 (both timepoints consisted of 1.5 g.kg^−1^ maltodextrin added to 600 mL of water and 150 mL of sugar free cordial). In this way, the timing and total dietary intake of protein (40 g), fat (16 g) and fluid ingestion (2.7 L) was matched between all 3 experimental trials though participants consumed a total of approximately 5–6 g.kg^−1^ CHO when completing the TRAIN HIGH trial (administered as maltodextrin, supplied by Science in Sport, UK; sugar free cordial was manufactured by Robinsons, UK).

### Quantification of training load

Pitch based training load was assessed using global positioning system (GPS) technology (Vector, Catapult, Melbourne, Australia). Each player was provided with a GPS unit (81 mm × 43 mm x 16 mm), accompanying heart rate monitor (Polar, UK) and custom-made manufacturer provided vest (Catapult, Melbourne, Australia) to wear on the upper back between both scapulae during each pitch-based training session. Each unit was alarmed to turn on thirty minutes prior to the start of each session to sample total distance (m), high speed running meters (> 5.5 m.s^−1^), meters per minute (m.min^−1^), accelerations (> 3 m.s^−1^), and decelerations (< 3 m.s^−1^) at 10 Hz providing a valid and reliable assessment of soccer specific movement (Coutts and Duffield 2010; Varley et al. 2012). To ascertain when academy soccer players are capable of achieving the training and match intensities of adult EPL players, absolute speed thresholds commonly used within the adult game were deliberately selected (Anderson et al. 2016b; Malone et al. 2015). Participants also reported their pre- and post-training assessment of ratings of perceived exertion (RPE 6 – 20) (Borg 1982), within minutes of the commencement and completion of the training sessions.

### Blood collection and analysis

Five millilitres of venous blood was drawn into one ethylenediaminetetraacetic acid (EDTA) tube (BD Vacutainer) and kept on ice until centrifugation at 1200 g for 10 min at 4 °C. A second five millilitre blood sample was collected into a serum tube and allowed to clot at room temperature for sixty minutes, before being centrifuged for 10 min at 1200 g at 4 °C. Following centrifugation, aliquots of plasma and serum were stored in eppendorfs at − 80 °C for subsequent analysis of plasma C-terminal telopeptide of type 1 collagen (βCTX), procollagen type I N Propeptide (PINP) and parathyroid hormone (PTH), and serum calcium (Ca), albumin and albumin adjusted calcium (ACa). These markers of bone resorption and formation can be released during bone remodelling and are, therefore, thought to reflect bone remodelling activity, with some suggestions that their measurement in blood can be useful in assessing bone turnover, and downstream prediction of bone loss (Vasikaran 2018). Fluctuations in protein concentrations, especially albumin, can cause total Ca concentrations to change independently of the ionized calcium concentration, as such Ca concentrations were adjusted against albumin concentrations to give an albumin-adjusted calcium (ACa) value using the following equation: ACa = [total calcium] + 0.02 × (40-[albumin]). Analysis of βCTX, PINP, PTH, Ca and ACa were performed at the Bioanalytical Facility, University of East Anglia by on a fully automated COBAS e601 system (Roche Diagnostics, Mannheim, Germany). βCTX, PINP and PTH were measured using electro-chemiluminescence immunoassay (ECLIA); kit# 09005773190, 03141071190 and 11,972,103,122, respectively. Quality controls (QC) were tested with each batch of samples; the inter-assay coefficient of variation (CV) for βCTX (n = 8) was ≤ 3% between 0.2 and 1.5 μg/L with the sensitivity of 0.01 μg/L; Inter-assay CV for PINP (n = 8) was < 3% between 20–600 µg/L with a sensitivity of 8 µg/L_._ the inter-assay CV for PTH (n = 8) was ≤ 3.8% across the analytical range of 0.127–530 pmoL. Total calcium and albumin were measured COBAS c501 system (Roche) by spectrophotometric methods; kit# 05061482190 and 03183688, respectively. The inter-assay CV (n = 8) for Ca was ≤ 1.6%, albumin was ≤ 1.1%.βCTX, PINP, PTH, Ca and ACa were selected for use as they are the preferred markers to assess the calcium homeostasis and bone turnover status in clinical studies (Vasikaran et al. 2011).

## Statistical analysis

All data were initially assessed for normality of distribution using the Shapiro–Wilk test. Comparisons between trials in training load metrics between TRAIN HIGH and TRAIN LOW trials were assessed using students t-tests for paired samples, where ninety-five percent confidence intervals (95% CI) for the differences are also presented. Comparisons of bone turnover markers and calcium metabolism between trials were assessed using a within subjects repeated measures general linear model where the within factors were time (*i.e.,* blood samples collected at 08:30, 10:30, 12:30 and 14:30) and trial (*i.e.,* REST, TRAIN LOW and TRAIN HIGH). Where significant main effects were present, Bonferroni post hoc analysis was conducted to locate specific differences and 95% CI for the differences are also presented where appropriate. All data in text, tables and figures are expressed as means and SD with *P* < 0.05 indicating statistical significance. Statistical tests were performed using SPSS for Windows (version 29, SPSS Inc, Chicago, IL).

## Results

### Training with reduced CHO availability does not affect training volume and intensity

The external and internal training load metrics of participants while training in TRAIN HIGH and TRAIN LOW conditions are presented in Table 2. No significant differences were apparent for total distance (*P* = 0.88), average speed (*P* = 0.56), high speed running distance (*P* = 0.72), number of accelerations (*P* = 0.65) and decelerations (*P* = 0.72). There was also no significant difference for average heart rate (*P* = 0.62) or post-session RPE (*P* = 0.96). When taken together, such data demonstrate that CHO availability did not affect the intensity and volume of training, therefore confirming that the acute training stimulus was comparable when players completed training in both TRAIN HIGH and TRAIN LOW conditions.

**Table 2:** An overview of external and internal training metrics for the TRAIN HIGH and TRAIN LOW trials

	TRAIN HIGH	TRAIN LOW	95% CI
Total distance (km)	5.6 ± 0.8 (4.2–6.8)	5.5 ± 1.1 (2.7–6.8)	− 1012 to 1067
Average speed (m.min^−1^)	81 ± 9 (70–95)	85 ± 12 (59–99)	− 15 to 9
High speed running (m)	350 ± 239 (33–533)	270 ± 89 (127–407)	− 148 to 202
Accelerations (n)	40 ± 12 (18–58)	44 ± 13 (15–62)	− 18 to 12
Decelerations (n)	38 ± 14 (7–55)	40 ± 12 (12–60)	− 15 to 12
Heart rate (bpm)	139 ± 10 (127–158)	142 ± 10 (127–158)	− 15 to 9
Post-session RPE	13 ± 3 (8–16)	14 ± 2 (10–16)	− 3 to 0

Data are presented as means with ± SD with range displayed in parentheses

### Completing an acute soccer-specific training with reduced CHO availability increases markers of bone resorption and formation

#### βCTX

**Fig. 2:**
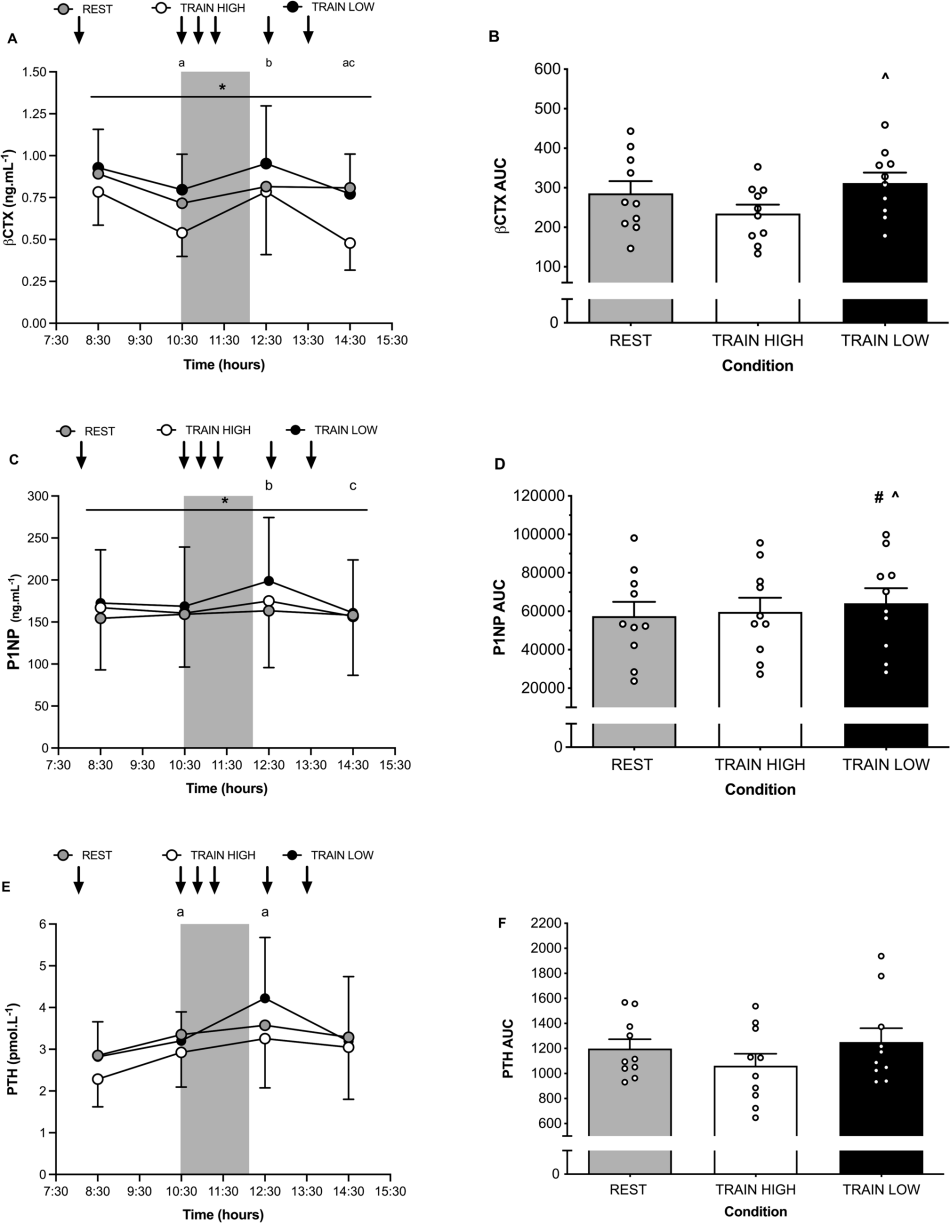
Plasma βCTX (**A**), PINP (**C**), PTH (**E**) concentrations before, during and after training. Shaded grey area denotes pitch-based training, downward arrows denote timing of feeding. Total area under the curve (AUC) for βCTX (**B**), PINP (**D**) and PTH (**F**) is also shown. ^*^ Denotes significant main effect for difference between conditions, ^a^denotes significant pairwise comparison difference from 08:30, ^b^denotes significant difference from 10:30 and ^c^denotes significant difference from 12:30, all* P* < 0.05; ^denotes significant difference in AUC between TRAIN LOW and TRAIN HIGH, # denotes significant difference in AUC between TRAIN LOW and REST, all* P* < 0.05. Grey, white and black bars represent mean data, individual data points are shown by white circles

As a marker of bone resorption, βCTX displayed significant main effects for time (*P* = 0.02), condition (*P* < 0.01) and interaction (*P* = 0.03) (see Fig. 2A). In relation to effects of time, pairwise comparisons demonstrate βCTX was significantly lower at 10:30 (*P* = 0.01) and 14:30 (*P* = 0.02) compared with 08:30. Such data suggest that nutrient ingestion in all three trials may have a role in reducing circulating βCTX. Furthermore, βCTX was significantly greater at 12:30 compared with both 10:30 (*P* = 0.018) and 14:30 (*P* < 0.01), thus suggesting that the acute training session significantly increased βCTX.

When considering pairwise comparisons for main effects of condition, βCTX was significantly lower in TRAIN HIGH compared with both TRAIN LOW (*P* < 0.01; 95% CI: -0.32 to -0.11 ng.mL^−1^) and REST (*P* = 0.04; 95% CI: -0.31 to -0.01 ng.mL^−1^), though no difference was apparent between TRAIN LOW and REST (*P* = 0.53; 95% CI: -0.06 to 0.17 ng.mL^−1^). Accordingly, the AUC for βCTX (see Fig. 2B) was significantly greater in TRAIN LOW compared with TRAIN HIGH (*P* < 0.01) while differences between TRAIN HIGH and REST were not significantly different (*P* = 0.07).

#### PINP

As a marker of bone formation, PINP displayed significant main effects for time (*P* < 0.01), condition (*P* < 0.01) and interaction (*P* < 0.01) (see Fig. 2C). In relation to effects of time, pairwise comparisons demonstrate PINP was significantly greater at 12:30 compared with both 10:30 (*P* = 0.01) and 14:30 (*P* < 0.01).

When considering pairwise comparisons for main effects of condition, TRAIN LOW was significantly greater than REST (*P* = 0.02; 95% CI: 2.8 to 30.3 ng.mL^−1^) yet there was no significant difference to TRAIN HIGH (*P* = 0.08; 95% CI: -1.3 to 22.2 ng.mL^−1^) and no difference was apparent between REST and TRAIN HIGH (*P* = 0.87; 95% CI: -21.9 to 9.8 ng.mL^−1^). In relation to AUC data (see Fig. 2D), TRAIN LOW was significantly greater than both TRAIN HIGH (*P* = 0.03) and REST (*P* = 0.01), though no difference was apparent between REST and TRAIN HIGH (*P* = 0.810).

### Completing an acute soccer-specific training with reduced CHO availability does not affect calcium metabolism

#### PTH

Changes in plasma PTH are presented in Fig. 2E. There was a significant main effect for time (*P* < 0.01), but no effect of condition (*P* = 0.14) or interaction effect (*P* = 0.32). In relation to pairwise comparisons for main effect of time, PTH was significantly greater at 10:30 (*P* = 0.02) and 12:30 (*P* < 0.01) compared with 08:30. Differences between 12:30 and 10:30 were not significantly different (*P* = 0.09). In accordance with no main effects for condition, the AUC also did not differ (*P* = 0.11) between trials (see Fig. 2F). These data suggest that the metabolic effects of acute feeding at breakfast and/or acute soccer-specific training is sufficient to increase PTH.

#### Calcium

**Fig. 3:**
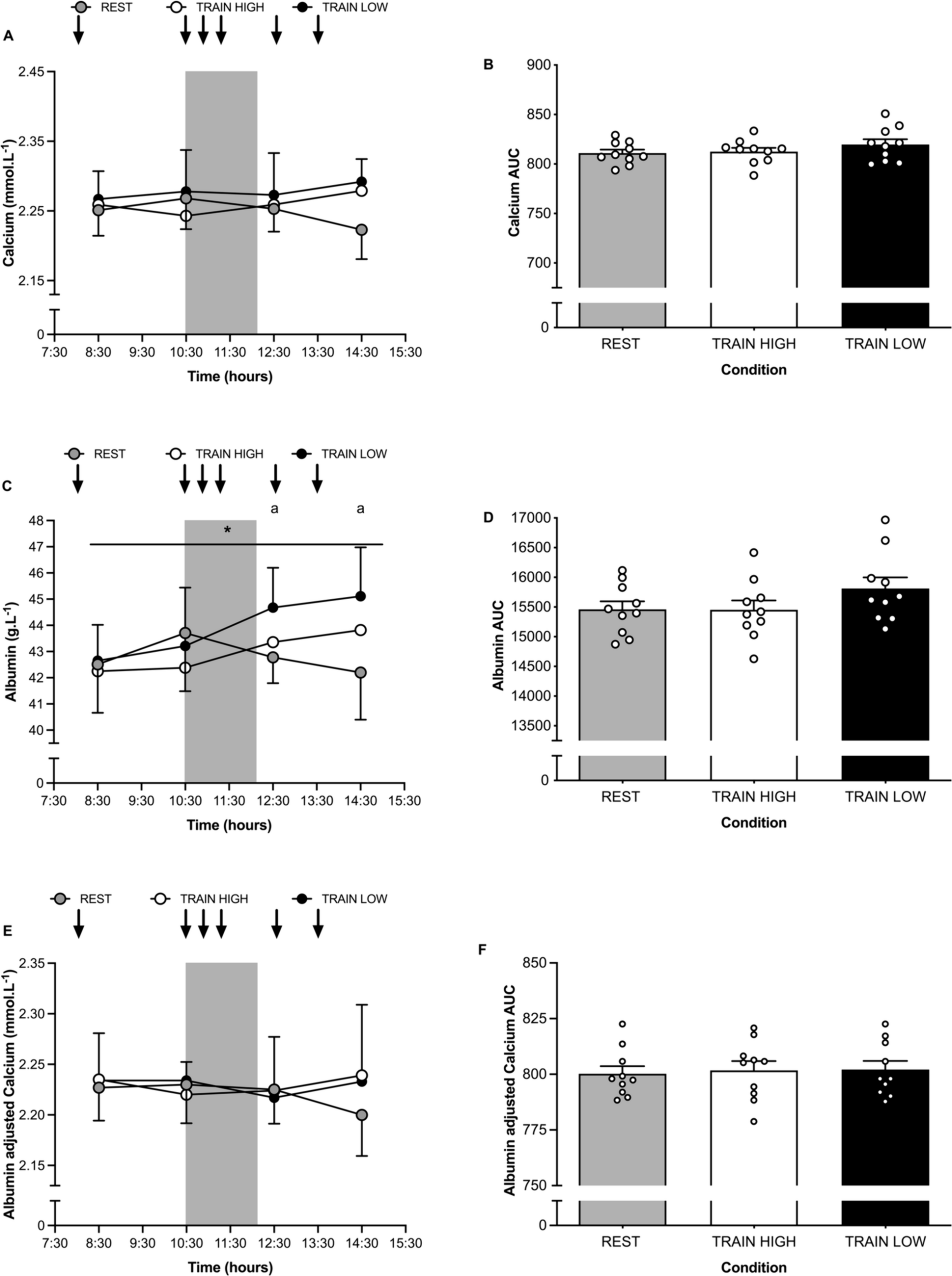
Serum calcium (**A**), albumin (**C**) and albumin adjusted calcium (**E**) concentrations before, during and after training. Shaded grey area denotes pitch-based training, downward arrows denote timing of feeding. Total area under the curve (AUC) for calcium (**B**), albumin (**D**) and albumin adjusted calcium (**F**) is also shown. ^*^ Denotes significant main effect for difference between conditions, ^a^denotes significant pairwise comparison difference from 08:30, all* P* < 0.05. Grey, white and black bars represent mean data, individual data points are shown by white circles

Changes in serum calcium are presented in Fig. 3A. There were no main effects of time (*P* = 0.91), condition (*P* = 0.20), or interaction (*P* = 0.07). In accordance, the AUC (see Fig. 3B) was also not significantly different between conditions (*P* = 0.30).

#### Albumin

Changes in serum albumin are presented in Fig. 3C. There was a significant main effect for time (*P* < 0.01), condition (*P* = 0.02) and an interaction effect (*P* < 0.01). In relation to pairwise comparisons for main effect of time, serum albumin was significantly different at 12:30 and 14:30 compared with 08:30 (both *P* < 0.01). In considering effects of condition, REST was significantly lower compared with TRAIN LOW (*P* = 0.03; 95% CI: -2.1 to – 0.1 g.L^−1^), though no differences were apparent between REST and TRAIN HIGH (*P* = 1.0; 95% CI: -1.2 to 0.9 g.L^−1^) or between TRAIN LOW and TRAIN HIGH (*P* = 0.21; 95% CI: -0.4 to 2.3 g.L^−1^). In relation to AUC data (see Fig. 3D), differences between conditions did not achieve statistical significance (*P* = 0.05).

#### Albumin adjusted calcium

Changes in albumin adjusted calcium are presented in Fig. 3E. There were no main effects for time (*P* = 0.59), condition (*P* = 0.67) or interaction (*P* = 0.23). In relation to AUC data (see Fig. 3F), there was no significant difference between conditions (*P* = 0.89).

## Discussion

In confirming our hypothesis, the present data demonstrate that completing an acute soccer-specific training session with reduced CHO availability increases bone resorption in academy soccer players. However, in contrast with our hypothesis, we also report that training with reduced CHO availability increases bone formation markers. Such alterations to markers of bone remodelling also occurred independent to changes in markers of calcium metabolism. Although the chronic implications of such acute fluctuations in bone (re)modelling markers could not be determined, it is possible that the combination of sub-optimal CHO intakes and high daily training volumes may in part, contribute to an increased risk of bone stress related injury and compromise bone development during growth and maturation. This assertion is especially relevant to the present population given the high daily energy demands associated with formalised training programmes, a culture of under-fuelling, and the prevalence of growth-related injuries.

Both longitudinal (Varley et al. 2023) and cross-sectional (Hagman et al. 2018) studies using bone imaging demonstrate that the loading stimulus associated with soccer training is anabolic to bone. It has also been reported that the loading stimulus induced by 12 weeks of soccer-specific training in academy players (with similar chronological age as the present cohort) was sufficient to induce increased tibial bone mass and density (Varley et al. 2017), whereas the training stimulus completed by a control group of recreational soccer players (*i.e.,* not enrolled on a formalised academy coaching programme) did not induce any detectable changes in bone characteristics (Varley et al. 2023). The present study extends our understanding of bone responses to soccer training by representing the first attempt to evaluate the acute bone response of markers of bone resorption and formation and calcium metabolism responses induced by an acute soccer training session in male players. Indeed, the ecological validity of our experimental model is strengthened by utilising a “real world” training session involving a field-based training session, as opposed to laboratory-based exercise. We also evaluated the role of CHO availability in modulating bone resorption and formation markers by utilising a repeated measures crossover design whereby players completed the session in conditions considered as best practice nutrition (Collins et al. 2021) or those indicative of the sub-optimal fuelling practices (*i.e.,* CHO restriction before, during and after training) previously reported by our group (Stables et al. 2022). Importantly, no significant differences were apparent in external and internal training load metrics between trials (see Table 1), thus suggesting that the training stimulus (*i.e.,* mechanical load) was likely similar between TRAIN HIGH and TRAIN LOW trials.

As an accepted marker of bone resorption, it is now well documented that βCTX is sensitive to the acute effects of both feeding and exercise (Walsh and Henriksen 2010). Notwithstanding the circadian variation of this marker (Bjarnason et al. 2002; Christgau 2000), the data presented here is in agreement with previous literature (Clowes et al. 2002) in considering that we observed that consumption of “breakfast” in all three trials significantly reduced βCTX concentrations in the two-hour postprandial period (see Fig. 2A), where the magnitude of reduction was more pronounced when CHO had been consumed in the TRAIN HIGH trial. In accordance with the effects of acute exercise (Dolan et al. 2022), completion of the acute soccer training session subsequently increased βCTX, although the effects of CHO feeding before and during the TRAIN HIGH trial ensured that absolute βCTX concentrations remained suppressed when compared with the TRAIN LOW trial. Similar to the effects of feeding at breakfast, post-training nutrient intake (*i.e.,* lunch) also caused a reduction in βCTX where again, the consumption of CHO in the TRAIN HIGH trial caused a greater magnitude of reduction. When taken together, such data clearly demonstrate that CHO feeding reduces βCTX concentrations (even in the presence of a high-intensity training stimulus) compared to training without CHO intake pre-, during and post training. We acknowledge, however, that future studies with greater access to elite participants and the potential for a greater sampling frequency should also obtain a fasted true baseline blood sample with additional sampling in the hours post-training to better understand changes in bone (re)modelling markers in the hours after pitch-based training. The relatively small number of samples which were obtained due to the nature of participants in this study may be considered a limitation to this study.

As a marker of bone formation, exercise-induced changes in PINP are less responsive than changes in βCTX, owing to the temporal processes underpinning bone resorption and formation whereby the basic multicellular unit is activated by an initial increase in bone resorption such that changes in bone formation would lag that of bone resorption (Dolan et al. 2020). Given the greater degree of uncoupled and site-specific bone modelling that occurs in adolescence (in addition to (re)modelling during skeletal growth in adolescence), it should also be noted that bone formation markers will be higher within this population as players develop peak bone mass (Seeman and Delmas 2006). The compounding impact upon these acute changes however would likely be negated given that the average age of participants in this study (17.4 ± 0.8 years) would be at the time whereby academy soccer players typically approach full skeletal maturity (Johnson et al. 2017) and a number of years post-PHV when growth rate would be highest during adolescence (Philippaerts et al. 2006). Previous research showed significant increases in PINP in adult males immediately after 60 min of treadmill running at 65% VO_2max_ (Scott et al. 2012), the magnitude of which was not affected if the exercise was performed fasted or fed (as achieved by a standardised breakfast of approximately 80 g CHO, 20 g fat, 10 g protein and 116 mg calcium). In contrast, Sale et al. (2015) later reported that the exercise-induced increases in PINP (also in adult males) immediately after 120 min of running at 70% VO_2max_ was significantly reduced when CHO was ingested during exercise at a rate of 0.7 g.kg^−1^ per hour (equivalent to approximately 50 g.h^−1^). Such data appear to agree with the present study given that we also observed significant increases in PINP at 12:30 (*i.e.,* post-training) when compared with the baseline sample at 08:30 (see Fig. 2C) and also when considering the fact that the AUC for PINP was significantly greater in TRAIN LOW versus both TRAIN HIGH and REST (see Fig. 2D). Although there is debate within the literature (Dolan et al. 2022) as to the physiological significance of such small and transient increases in PINP (*i.e.,* such short-term timescales may not be representative of true exercise-induced increase in collagen deposition), it is noteworthy that the model of acute CHO restriction used here increased both bone (re)modelling markers βCTX and PINP. In this way, evaluation of the temporal responses of PINP across the sampling period are suggestive of the possibility that such elevated PINP responses in TRAIN LOW may occur as a compensatory response to the earlier challenge of CHO (and energy) restriction at breakfast and during exercise that has already presented as acute increases in bone resorption markers, which would otherwise be attenuated in the TRAIN HIGH trial due to greater CHO and energy availability. As such, the early and later responses of βCTX and PINP, may indeed represent an acute physiological adjustment to rates of bone remodelling markers to try and maintain the dynamic balance between bone resorption and formation in the face of the physiological challenge of both CHO restriction and high-intensity exercise. Such data suggest that under conditions of low CHO availability, acute soccer-specific training significantly increases PINP, yet it was not possible to determine the physiological relevance of such an acute change to bone tissue. To that end, it should be noted that βCTX and PINP are not specific to bone tissue and the increase observed here could also reflect leakage from connective tissue or collagen metabolism from other tissues (Vasikaran et al. 2011).

The mechanisms by which manipulation of CHO availability before, during and after exercise affects exercise-induced alterations in markers of bone resorption and formation are not yet well understood. However, in agreement with previous researchers (Scott et al. 2012; Sale et al. 2015), we also observed that CHO restriction did not affect albumin adjusted calcium (see Fig. 3A, B, E and F) or exercise-induced increases in PTH (see Fig. 2E and F). Although we acknowledge that our frequency of sampling did not allow for evaluation of calcium metabolism during exercise (nor the ability to measure free calcium due to the limitations of testing in the elite athlete environment, particularly as this pertains to the required timeframe of sampling), our data are in support of the hypothesis that CHO likely regulates exercise-induced bone resorption and formation through pathways not related to calcium metabolism. Rather, it is possible that the provision of nutrient intake before exercise causes an initial reduction in bone resorption that is mediated, in part, through the gut derived incretin hormones of glucose-dependent insulinotropic polypeptide (GIP) and glucagon-like peptide 1 (GLP-1) (Bergmann et al. 2019). The combination of CHO restriction before and during high-intensity exercise may also facilitate cross-talk between muscle, adipocytes and bone (Kirk et al. 2020), as facilitated through the action of key myokine and adipokines such as interleukin 6 and leptin. While outside the scope of this work, evidence in support for a modulatory role of interleukin-6 (IL-6) in regulation of acute markers of bone metabolism is provided from several studies. For example, when exercising in conditions of CHO restriction (Heikura et al. 2019) or with low muscle glycogen (Keller et al. 2001; Steensberg et al. 2001), release of muscle derived IL-6 (Febbraio and Pedersen 2002) and circulating IL-6 concentrations (Starkie et al. 2001) are augmented compared to when CHO has been ingested before and/or during exercise. In such situations, IL-6 is thought to act in an endocrine like action upon the liver to maintain glucose homeostasis (Pedersen and Febbraio 2008). However, its effect on bone may be less favourable and indeed, evidence from in vitro and animal models collectively demonstrate that IL-6, in the presence of soluble IL-6 receptors, can stimulate osteoclastogenesis and a net resorptive effect (Kirk et al. 2020). Interestingly, Sale et al. (2015) previously observed a significant correlation between exercise-induced changes in IL-6 and βCTX, thus providing further evidence in support of a mechanistic link between muscle and bone under the physiological stress of CHO restriction and exercise. In addition, we previously observed in a similar model of CHO (and energy) restriction to that studied here (*i.e.,* restriction of CHO intake before, during and after 1 h of high-intensity intermittent running) that exercise completed with reduced CHO availability significantly augmented both IL-6 and leptin concentrations immediately post- and at 3 h post-exercise compared with exercise completed in conditions where CHO had been fed before (3 g.kg^−1^), during (60 g) and after (4 g.kg^−1^) exercise (Hammond et al. 2019). Nonetheless, we acknowledge the limitation that our sampling volume and frequency did not allow us to assess a broader range of bone markers alongside myokine, osteokine and adipokine related signalling, including both blood glucose and insulin. Notably glucose and insulin have been shown to acutely attenuate bone resorption (Sherk et al. 2020), which may provide further context for the results of this study. Indeed while the bone resorption and formation markers employed within this study are reference bone markers and biochemical by-products of osteoblast activity (Vasikaran et al. 2011), there remains no bone marker that reflects the bone remodelling process with perfect specificity and sensitivity (Vasikaran 2018). Further studies are now required to provide a more rigorous assessment of the mechanisms by which CHO restriction and exercise may regulate bone resorption.

It is thought that an initial transient period of bone catabolism is necessary to stimulate the bone remodelling cycle (Robling et al. 2006; Dolan et al. 2020) and hence, an initial increase in exercise-induced bone resorption provides the stimulus to subsequently increase bone formation. In this regard, our data could be interpreted to support the anabolic potential of soccer training for bone, given that the training session completed here was sufficient to initiate the acute bone remodelling process. However, if the process of resorption is left unchecked (as stimulated by high daily training volumes and sub-optimal CHO and energy intake *i.e.,* TRAIN LOW conditions), this may favour bone resorption. Such a model has been suggested to play a contributory role in mediating the low bone mineral density in road cyclists (Hilkens et al. 2023) and of note, most prevalent injury that occurs in academy players during the times of peak height velocity has been reported as growth related injuries to the lower back, sacrum, pelvis, and knee (Hall et al. 2020). Although there is a potential theoretical benefits (e.g. cell signalling regulating oxidative adaptions) for consuming reduced CHO intake in relation to aerobic type training (Bartlett et al. 2015; Impey et al. 2016), our data further demonstrate that athletes who wish to regularly train in a state of reduced CHO availability should be aware of potential negative effects upon bone. This of specific relevance to adolescent athletes and the present data provide further justification that CHO restriction should not be practiced in athletic populations who are not yet physically mature.

In summary, the present study provides the first report to characterise the effects of an acute soccer-specific training session on markers of bone resorption, bone formation and calcium metabolism in male academy soccer players. Importantly, our data demonstrate that soccer training increases bone (re)modelling makers and that training with reduced CHO availability augments bone resorption and formation markers. While an increase in bone formation markers may be seen as anabolic to bone, it is suggested that the commonly reported sub-optimal fuelling practices of academy players (as replicated in the present experimental design) and apparent increase in bone resorption markers may impair skeletal development during growth and maturation as players transition through the academy development pathway. Further studies are now required to ascertain the mechanisms by which training with CHO availability regulates bone resorption. Given the growing body of literature demonstrating that CHO availability affects exercise-induced bone remodelling markers, our data also suggest that the benefits of CHO should be communicated to players and stakeholders over and above that of physical and technical performance.

## Data Availability

Anonymised data may be made available upon request.

## Abbreviations

ACa: Albumin adjusted calcium
AUC: Area under the curve
βCTX: β-Carboxyterminal telopeptide
Ca: Calcium
CHO: Carbohydrate
EDTA: Ethylenediaminetetraacetic acid
EPL: English Premier League
FP: Foundation phase
GIP: Glucose-dependent insulinotropic polypeptide
GLP-1: Glucagon-like peptide 1
GPS: Global positioning system
IL-6: Interleukin-6
LEA: Low energy availability
PTH: Parathyroid hormone
P1NP: Procollagen-1 N-terminal peptide
PDP: Professional development phase
YDP: Youth development phase

## References

[CR1] Anderson L, Orme P, Di Michele R, Close GL, Morgans R, Drust B, Morton JP (2016a) Quantification of training load during one-, two- and three-game week schedules in professional soccer players from the English Premier League: implications for carbohydrate periodisation. J Sports Sci 34(13):1250–1259. 10.1080/02640414.2015.1106574 26536538 10.1080/02640414.2015.1106574

[CR2] Anderson L, Orme P, Michele RD, Close GL, Milsom J, Morgans R, Drust B, Morton JP (2016) Quantification of seasonal-long physical load in soccer players with different starting status from the English Premier League: implications for maintaining squad physical fitness. IJSPP. 11(8):1038–1046. 10.1123/ijspp.2015-0672 26915393 10.1123/ijspp.2015-0672

[CR3] Anderson L, Orme P, Naughton RJ, Close GL, Milsom J, Rydings D, O’Boyle A, Di Michele R, Louis J, Hambly C, Speakman JR, Morgans R, Drust B, Morton JP (2017) energy intake and expenditure of professional soccer players of the English Premier League: evidence of carbohydrate periodization. Int J Sport Nutr Exerc Metab 27(3):228–238. 10.1123/ijsnem.2016-0259 28050927 10.1123/ijsnem.2016-0259

[CR4] Bartlett JD, Hawley JA, Morton JP (2015) Carbohydrate availability and exercise training adaptation: too much of a good thing? Eur J Sport Sci 15(1):3–12. 10.1080/17461391.2014.920926 24942068 10.1080/17461391.2014.920926

[CR5] Bergmann NC, Lund A, Gasbjerg LS, Jørgensen NR, Jessen L, Hartmann B, Holst JJ, Christensen MB, Vilsbøll T, Knop FK (2019) Separate and combined effects of GIP and GLP-1 infusions on bone metabolism in overweight men without diabetes. The J Clin Endocrinol Metab 104(7):2953–2960. 10.1210/jc.2019-00008 30848791 10.1210/jc.2019-00008

[CR6] Bjarnason NH, Henriksen EE, Alexandersen P, Christgau S, Henriksen DB, Christiansen C (2002) Mechanism of circadian variation in bone resorption. Bone 30(1):307–313. 10.1016/s8756-3282(01)00662-7 11792602 10.1016/s8756-3282(01)00662-7

[CR7] Borg GA (1982) Psychophysical bases of perceived exertion. Med Sci Sports Exerc 14(5):377–381 7154893

[CR8] Brownlee TE, O’Boyle A, Morgans R, Morton JP, Erskine RM, Drust B (2018) Training duration may not be a predisposing factor in potential maladaptations in talent development programmes that promote early specialisation in elite youth soccer. Int J Sports Sci Coach 13(5):674–678. 10.1177/1747954117752127

[CR9] Carter JL, Lee DJ, Ranchordas MK, Cole M (2022) Perspectives of the barriers and enablers to nutritional adherence in professional male academy football players. Sci Med Footb. 10.1080/24733938.2022.2123554 36082957 10.1080/24733938.2022.2123554

[CR10] Christgau S (2000) Circadian variation in serum CrossLaps concentration is reduced in fasting individuals. Clin Chem 46(3):431 10702538

[CR11] Clowes JA, Hannon RA, Yap TS, Hoyle NR, Blumsohn A, Eastell R (2002) Effect of feeding on bone turnover markers and its impact on biological variability of measurements. Bone 30(6):886–890. 10.1016/s8756-3282(02)00728-7 12052458 10.1016/s8756-3282(02)00728-7

[CR12] Collins J, Maughan RJ, Gleeson M, Bilsborough J, Jeukendrup A, Morton JP, Phillips SM, Armstrong L, Burke LM, Close GL, Duffield R, Larson-Meyer E, Louis J, Medina D, Meyer F, Rollo I, Sundgot-Borgen J, Wall BT, Boullosa B, Dupont G, Lizarraga A, Res P, Bizzini M, Castagna C, Cowie CM, D’Hooghe M, Geyer H, Meyer T, Papadimitriou N, Vouillamoz M, McCall A (2021) UEFA expert group statement on nutrition in elite football. current evidence to inform practical recommendations and guide future research. Br J Sports Med 55(8):416. 10.1136/bjsports-2019-101961 33097528 10.1136/bjsports-2019-101961

[CR13] Coutts AJ, Duffield R (2010) Validity and reliability of GPS devices for measuring movement demands of team sports. J Sci Med Sport 13(1):133–135. 10.1016/j.jsams.2008.09.015 19054711 10.1016/j.jsams.2008.09.015

[CR14] de Sousa MV, Pereira RMR, Fukui R, Caparbo VF, da Silva MER (2014) Carbohydrate beverages attenuate bone resorption markers in elite runners. Metab 63(12):1536–1541. 10.1016/j.metabol.2014.08.011 10.1016/j.metabol.2014.08.011 25239216

[CR15] Dolan E, Varley I, Ackerman KE, Pereira RMR, Elliott-Sale KJ, Sale C (2020) The bone metabolic response to exercise and nutrition. Exerc Sport Sci Rev 48(2):49–58. 10.1249/jes.0000000000000215 31913188 10.1249/JES.0000000000000215

[CR16] Dolan E, Dumas A, Keane KM, Bestetti G, Freitas LHM, Gualano B, Kohrt WM, Kelley GA, Pereira RMR, Sale C, Swinton PA (2022) The bone biomarker response to an acute bout of exercise: a systematic review with meta-analysis. Sports Med 52(12):2889–2908. 10.1007/s40279-022-01718-8 35870108 10.1007/s40279-022-01718-8

[CR17] Febbraio MA, Pedersen BK (2002) Muscle-derived interleukin-6: mechanisms for activation and possible biological roles. Faseb j 16(11):1335–1347. 10.1096/fj.01-0876rev 12205025 10.1096/fj.01-0876rev

[CR18] Fensham NC, Heikura IA, McKay AKA, Tee N, Ackerman KE, Burke LM (2022) Short-Term carbohydrate restriction impairs bone formation at rest and during prolonged exercise to a greater degree than low energy availability. JMBR 37(10):1915–1925. 10.1002/jbmr.4658 10.1002/jbmr.4658 PMC9804216 35869933

[CR19] Goulding A (2007) Risk factors for fractures in normally active children and adolescents. Med Sport Sci 51:102–120. 10.1159/000103007 17505122 10.1159/000103007

[CR20] Hagman M, Helge EW, Hornstrup T, Fristrup B, Nielsen JJ, Jørgensen NR, Andersen JL, Helge JW, Krustrup P (2018) Bone mineral density in lifelong trained male football players compared with young and elderly untrained men. J Sport and Health Sci 7(2):159–168. 10.1016/j.jshs.2017.09.009 30356456 10.1016/j.jshs.2017.09.009 PMC6180542

[CR21] Hall ECR, Larruskain J, Gil SM, Lekue JA, Baumert P, Rienzi E, Moreno S, Tannure M, Murtagh CF, Ade JD, Squires P, Orme P, Anderson L, Whitworth-Turner CM, Morton JP, Drust B, Williams AG, Erskine RM (2020) An injury audit in high-level male youth soccer players from English, Spanish, Uruguayan and Brazilian academies. Phys Ther Sport 44:53–60. 10.1016/j.ptsp.2020.04.033 32416582 10.1016/j.ptsp.2020.04.033

[CR22] Hammond KM, Sale C, Fraser W, Tang J, Shepherd SO, Strauss JA, Close GL, Cocks M, Louis J, Pugh J, Stewart C, Sharples AP, Morton JP (2019) Post-exercise carbohydrate and energy availability induce independent effects on skeletal muscle cell signalling and bone turnover: implications for training adaptation. J Physiol 597(18):4779–4796. 10.1113/JP278209 31364768 10.1113/JP278209

[CR23] Hannon MP, Carney DJ, Floyd S, Parker LJF, McKeown J, Drust B, Unnithan VB, Close GL, Morton JP (2020) Cross-sectional comparison of body composition and resting metabolic rate in Premier League academy soccer players: Implications for growth and maturation. J Sports Sci 38(11–12):1326–1334. 10.1080/02640414.2020.1717286 31964230 10.1080/02640414.2020.1717286

[CR24] Hannon MP, Coleman NM, Parker LJF, McKeown J, Unnithan VB, Close GL, Drust B, Morton JP (2021a) Seasonal training and match load and micro-cycle periodization in male Premier League academy soccer players. J Sports Sci 39(16):1838–1849. 10.1080/02640414.2021.1899610 33759688 10.1080/02640414.2021.1899610

[CR25] Hannon MP, Parker LJF, Carney DJ, McKeown J, Speakman JR, Hambly C, Drust B, Unnithan VB, Close GL, Morton JP (2021b) Energy requirements of male academy soccer players from the English Premier League. Med Sci Sports Exerc 53(1):200–210. 10.1249/mss.0000000000002443 32701871 10.1249/MSS.0000000000002443

[CR26] Heikura IA, Burke LM, Hawley JA, Ross ML, Garvican-Lewis L, Sharma AP, McKay AKA, Leckey JJ, Welvaert M, McCall L, Ackerman KE (2019) A Short-term ketogenic diet impairs markers of bone health in response to exercise. Front Endocrinol (lausanne) 10:880. 10.3389/fendo.2019.00880 32038477 10.3389/fendo.2019.00880 PMC6985427

[CR27] Hilkens L, van Schijndel N, Weijer V, Boerboom M, van der Burg E, Peters V, Kempers R, Bons J, van Loon LJ, van Dijk J-W (2023) Low bone mineral density and associated risk factors in elite cyclists at different stages of a professional cycling career. Med Sci Sports Exerc 55(5):957 36595659 10.1249/MSS.0000000000003113 PMC10090358

[CR28] Hoenig T, Eissele J, Strahl A, Popp KL, Stürznickel J, Ackerman KE, Hollander K, Warden SJ, Frosch K-H, Tenforde AS, Rolvien T (2023) Return to sport following low-risk and high-risk bone stress injuries: a systematic review and meta-analysis. Br J Sports Med 57(7):427–432. 10.1136/bjsports-2022-106328 36720584 10.1136/bjsports-2022-106328

[CR29] Impey SG, Hammond KM, Shepherd SO, Sharples AP, Stewart C, Limb M, Smith K, Philp A, Jeromson S, Hamilton DL, Close GL, Morton JP (2016) Fuel for the work required: a practical approach to amalgamating train-low paradigms for endurance athletes. Physiol Rep. 4(10):e12803. 10.14814/phy2.12803 27225627 10.14814/phy2.12803 PMC4886170

[CR30] Johnson A, Farooq A, Whiteley R (2017) Skeletal maturation status is more strongly associated with academy selection than birth quarter. Sci Med Footb 1(2):157–163

[CR31] Keller C, Steensberg A, Pilegaard H, Osada T, Saltin B, Pedersen BK, Neufer PD (2001) Transcriptional activation of the IL-6 gene in human contracting skeletal muscle: influence of muscle glycogen content. FASEB J 15(14):1–15. 10.1096/fj.01-0507fje 10.1096/fj.01-0507fje 11687509

[CR32] Kirk B, Feehan J, Lombardi G, Duque G (2020) Muscle, bone, and fat crosstalk: the biological role of myokines, osteokines, and adipokines. Curr Osteoporos Rep 18(4):388–400. 10.1007/s11914-020-00599-y 32529456 10.1007/s11914-020-00599-y

[CR33] Malone JJ, Di Michele R, Morgans R, Burgess D, Morton JP, Drust B (2015) Seasonal training-load quantification in elite English Premier League soccer players. IJSPP 10(4):489–497. 10.1123/ijspp.2014-0352 25393111 10.1123/ijspp.2014-0352

[CR34] Mountjoy M, Ackerman KE, Bailey DM, Burke LM, Constantini N, Hackney AC, Heikura IA, Melin A, Pensgaard AM, Stellingwerff T, Sundgot-Borgen JK, Torstveit MK, Jacobsen AU, Verhagen E, Budgett R, Engebretsen L, Erdener U (2023) 2023 International Olympic Committee’s (IOC) consensus statement on Relative Energy Deficiency in Sport (REDs). Bri J Sports Med 57(17):1073–1097. 10.1136/bjsports-2023-106994 10.1136/bjsports-2023-106994 37752011

[CR35] Naughton RJ, Drust B, O’Boyle A, Morgans R, Abayomi J, Davies IG, Morton JP, Mahon E (2016) Daily distribution of carbohydrate, protein and fat intake in elite youth academy soccer players over a 7-day training period. Int J Sport Nutr Exerc Metab 26(5):473–480. 10.1123/ijsnem.2015-0340 27633998 10.1123/ijsnem.2015-0340

[CR36] Pedersen BK, Febbraio MA (2008) Muscle as an endocrine organ: focus on muscle-derived interleukin-6. Physiol Rev 88(4):1379–1406 18923185 10.1152/physrev.90100.2007

[CR37] Philippaerts RM, Vaeyens R, Janssens M, Van Renterghem B, Matthys D, Craen R, Bourgois J, Vrijens J, Beunen G, Malina RM (2006) The relationship between peak height velocity and physical performance in youth soccer players. J Sports Sci 24(3):221–230. 10.1080/02640410500189371 16368632 10.1080/02640410500189371

[CR38] Robling AG, Castillo AB, Turner CH (2006) Biomechanical and molecular regulation of bone remodeling. Annu Rev Biomed Eng 8:455–498. 10.1146/annurev.bioeng.8.061505.095721 16834564 10.1146/annurev.bioeng.8.061505.095721

[CR39] Sale C, Varley I, Jones TW, James RM, Tang JC, Fraser WD, Greeves JP (2015) Effect of carbohydrate feeding on the bone metabolic response to running. J Appl Physiol 119(7):824–830. 10.1152/japplphysiol.00241.2015 26251510 10.1152/japplphysiol.00241.2015 PMC4593812

[CR40] Scott JP, Sale C, Greeves JP, Casey A, Dutton J, Fraser WD (2012) Effect of fasting versus feeding on the bone metabolic response to running. Bone 51(6):990–999 22960044 10.1016/j.bone.2012.08.128

[CR41] Seeman E, Delmas PD (2006) Bone quality—the material and structural basis of bone strength and fragility. N Engl J Med 354(21):2250–2261 16723616 10.1056/NEJMra053077

[CR42] Sherk VD, Schauer I, Shah VN (2020) Update on the acute effects of glucose, insulin, and incretins on bone turnover in vivo. Curr Osteoporos Rep 18(4):371–377. 10.1007/s11914-020-00598-z 32504189 10.1007/s11914-020-00598-z PMC8118128

[CR43] Stables RG, Hannon MP, Costello NB, McHaffie SJ, Sodhi JS, Close GL, Morton JP (2022) Acute fuelling and recovery practices of academy soccer players: implications for growth, maturation, and physical performance. Sci Med Footb. 10.1080/24733938.2022.2146178 36351858 10.1080/24733938.2022.2146178

[CR44] Stables RG, Hannon MP, Jacob AD, Topping O, Costello NB, Boddy LM, Hambly C, Speakman JR, Sodhi JS, Close GL, Morton JP (2023) Daily energy requirements of male academy soccer players are greater than age-matched non-academy soccer players: a doubly labelled water investigation. J Sports Sci. 10.1080/02640414.2023.2263707 37811806 10.1080/02640414.2023.2263707

[CR45] Starkie RL, Arkinstall MJ, Koukoulas I, Hawley JA, Febbraio MA (2001) Carbohydrate ingestion attenuates the increase in plasma interleukin-6, but not skeletal muscle interleukin-6 mRNA, during exercise in humans. J Physiol 533(Pt 2):585–591. 10.1111/j.1469-7793.2001.0585a.x 11389214 10.1111/j.1469-7793.2001.0585a.x PMC2278645

[CR46] Steensberg A, Febbraio MA, Osada T, Schjerling P, van Hall G, Saltin B, Pedersen BK (2001) Interleukin-6 production in contracting human skeletal muscle is influenced by pre-exercise muscle glycogen content. J Physiol 537(Pt 2):633–639. 10.1111/j.1469-7793.2001.00633.x 11731593 10.1111/j.1469-7793.2001.00633.x PMC2278951

[CR47] Townsend R, Elliott-Sale KJ, Currell K, Tang J, Fraser WD, Sale C (2017) The effect of postexercise carbohydrate and protein ingestion on bone metabolism. Med Sci Sports Exerc 49(6):1209–1218. 10.1249/mss.0000000000001211 28121797 10.1249/MSS.0000000000001211

[CR48] Varley MC, Fairweather IH, Aughey RJ (2012) Validity and reliability of GPS for measuring instantaneous velocity during acceleration, deceleration, and constant motion. J Sports Sci 30(2):121–127. 10.1080/02640414.2011.627941 22122431 10.1080/02640414.2011.627941

[CR49] Varley I, Hughes DC, Greeves JP, Fraser WD, Sale C (2017) Increased training volume improves bone density and cortical area in adolescent football players. Int J Sports Med 38(05):341–346. 10.1055/s-0042-124510 28249346 10.1055/s-0042-124510

[CR50] Varley I, Sale C, Greeves JP, Morris JG, Sunderland C, Saward C (2023) Relationship between football-specific training characteristics and tibial bone adaptation in male academy football players. Sports (Basel) 11(4):86. 10.3390/sports11040086 37104160 10.3390/sports11040086 PMC10145492

[CR51] Vasikaran S (2018) Assessment of bone turnover in osteoporosis: harmonization of the total testing process. CCLM 56(10):1603–1607. 10.1515/cclm-2017-1109 29381471 10.1515/cclm-2017-1109

[CR52] Vasikaran S, Eastell R, Bruyère O, Foldes AJ, Garnero P, Griesmacher A, McClung M, Morris HA, Silverman S, Trenti T, Wahl DA, Cooper C, Kanis JA (2011) Markers of bone turnover for the prediction of fracture risk and monitoring of osteoporosis treatment: a need for international reference standards. Osteoporos Int 22(2):391–420. 10.1007/s00198-010-1501-1 21184054 10.1007/s00198-010-1501-1

[CR53] Walsh JS, Henriksen DB (2010) Feeding and bone. Arch Biochem Biophys 503(1):11–19. 10.1016/j.abb.2010.06.020 20599659 10.1016/j.abb.2010.06.020

[CR54] Wrigley R, Drust B, Stratton G, Scott M, Gregson W (2012) Quantification of the typical weekly in-season training load in elite junior soccer players. J Sports Sci 30(15):1573–1580. 10.1080/02640414.2012.709265 22852843 10.1080/02640414.2012.709265

[CR55] Zhang Y, Zhang J, Huang X, Yu X, Li Y, Yu F, Zhou W (2023) Variation of bone turnover markers in childhood and adolescence. Int J Clin Pract 2023:5537182. 10.1155/2023/5537182 37547099 10.1155/2023/5537182 PMC10403322

